# Associations between cardiorespiratory fitness and lifestyle‐related factors with DNA methylation‐based ageing clocks in older men: WASEDA'S Health Study

**DOI:** 10.1111/acel.13960

**Published:** 2023-08-16

**Authors:** Takuji Kawamura, Zsolt Radak, Hiroki Tabata, Hiroshi Akiyama, Nobuhiro Nakamura, Ryoko Kawakami, Tomoko Ito, Chiyoko Usui, Matyas Jokai, Ferenc Torma, Hyeon‐Ki Kim, Motohiko Miyachi, Suguru Torii, Katsuhiko Suzuki, Kaori Ishii, Shizuo Sakamoto, Koichiro Oka, Mitsuru Higuchi, Isao Muraoka, Kristen M. McGreevy, Steve Horvath, Kumpei Tanisawa

**Affiliations:** ^1^ Waseda Institute for Sport Sciences, Waseda University Saitama Japan; ^2^ Research Centre for Molecular Exercise Science Hungarian University of Sports Science Budapest Hungary; ^3^ Faculty of Sport Sciences Waseda University Saitama Japan; ^4^ Sportology Centre Juntendo University Graduate School of Medicine Tokyo Japan; ^5^ Graduate School of Sport Sciences Waseda University Saitama Japan; ^6^ Physical Fitness Research Institute, Meiji Yasuda Life Foundation of Health and Welfare Tokyo Japan; ^7^ Department of Food and Nutrition Tokyo Kasei University Tokyo Japan; ^8^ Faculty of Health and Sport Sciences University of Tsukuba Ibaraki Japan; ^9^ Faculty of Sport Science Surugadai University Saitama Japan; ^10^ Department of Biostatistics, Fielding School of Public Health University of California Los Angeles Los Angeles California USA; ^11^ Department of Human Genetics, David Geffen School of Medicine University of California Los Angeles Los Angeles California USA

**Keywords:** body composition, chronotype, drinking, elderly Japanese men, epigenetic clock, micronutrients, peak oxygen uptake, smoking

## Abstract

DNA methylation‐based age estimators (DNAm ageing clocks) are currently one of the most promising biomarkers for predicting biological age. However, the relationships between cardiorespiratory fitness (CRF), measured directly by expiratory gas analysis, and DNAm ageing clocks are largely unknown. We investigated the relationships between CRF and the age‐adjusted value from the residuals of the regression of DNAm ageing clock to chronological age (DNAmAgeAcceleration: DNAmAgeAccel) and attempted to determine the relative contribution of CRF to DNAmAgeAccel in the presence of other lifestyle factors. DNA samples from 144 Japanese men aged 65–72 years were used to appraise first‐ (i.e., DNAmHorvath and DNAmHannum) and second‐ (i.e., DNAmPhenoAge, DNAmGrimAge, and DNAmFitAge) generation DNAm ageing clocks. Various surveys and measurements were conducted, including physical fitness, body composition, blood biochemical parameters, nutrient intake, smoking, alcohol consumption, disease status, sleep status, and chronotype. Both oxygen uptake at ventilatory threshold (VO_2_/kg at VT) and peak oxygen uptake (VO_2_/kg at Peak) showed a significant negative correlation with GrimAgeAccel, even after adjustments for chronological age and smoking and drinking status. Notably, VO_2_/kg at VT and VO_2_/kg at Peak above the reference value were also associated with delayed GrimAgeAccel. Multiple regression analysis showed that calf circumference, serum triglyceride, carbohydrate intake, and smoking status, rather than CRF, contributed more to GrimAgeAccel and FitAgeAccel. In conclusion, although the contribution of CRF to GrimAgeAccel and FitAgeAccel is relatively low compared to lifestyle‐related factors such as smoking, the results suggest that the maintenance of CRF is associated with delayed biological ageing in older men.

## Graphical abstract

The contribution of cardiorespiratory fitness (CRF) to GrimAgeAcceleration and FitAgeAcceleration are relatively low compared to lifestyle‐related factors such as serum triglyceride, daily intake of carbohydrate, smoking, and calf circumference. There is a negative correlation between CRF and GrimAgeAcceleration, even after adjusting for chronological age, smoking, and alcohol consumption, and maintenance of CRF above reference values is negatively correlated with delayed GrimAgeAcceleration.

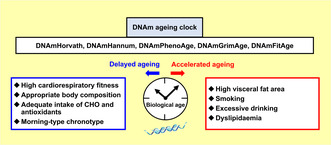

## 1 INTRODUCTION

Population ageing is an issue in many countries worldwide; in fact, between 2000 and 2019, the global life expectancy at birth increased by 6.5 years (World Health Organization, 2022). More importantly, there is a considerable gap of approximately 10 years between life expectancy and healthy life expectancy, regardless of the region and wealth of countries (World Health Organization, 2022). This gap is not limited to individual problems, such as a decline in quality of life, but is also directly linked to socioeconomic losses, such as increased medical costs and long‐term care burdens. To improve this current situation and extend health span, it is necessary to establish intervention strategies that target ageing itself, the strongest risk factor for each disease, rather than just focussing on organ‐ and disease‐based segmented medicine (Campisi et al., 2019). Chronological age, the number of years since birth, is frequently used as a measure of ageing but is often accompanied by significant interindividual variabilities in health outcomes among older adults (Lowsky et al., 2014). Therefore, ageing biomarkers that can predict biological age, which reflects the biological state of the individual, have been explored for over 30 years (Baker & Sprott, 1988). Developing such ageing surrogate biomarkers could be useful for assessing and validating anti‐ageing interventions on the rate of ageing in each individual within a relatively short time frame.

DNA methylation‐based age estimators (DNAm ageing clocks) are currently considered to be one of the most promising biomarkers for predicting biological age, as they have demonstrated higher predictive power for mortality in a certain number of studies compared to other biological age predictors, including telomere length (Jylhävä et al., 2017). DNAm ageing clocks are developed using a mathematical algorithm based on the methylation level of cytosine in the region where cytosine and guanine appear consecutively on the same strand of nucleic acid in the genome (CpG site). For example, the “first generation” DNAm ageing clocks, DNAmAgeHorvath, and DNAmAgeHannum, use methylation levels of 353 and 71 CpG sites, respectively, and they have been shown to correlate strongly with chronological age (Hannum et al., 2013; Horvath, 2013). The subsequently proposed “second generation” composite clocks, DNAmPhenoAge and DNAmGrimAge, use DNAm to predict non‐DNA traits, which can be used as additional variables to predict biological age, disease status, and mortality accurately (Levine et al., 2018; Lu et al., 2019; Simpson & Chandra, 2021). Recently, a novel composite biomarker, DNAmFitAge, that incorporates the physical fitness parameters of gait speed, hand grip strength, forced expiratory volume in 1 sec (FEV1), and maximal oxygen uptake (VO_2max_), has been developed (McGreevy et al., 2023). These DNAm‐based biomarkers are expressed as absolute values (DNAmAge) or age‐adjusted values from the residuals of the regression of each DNAm ageing clock to chronological age (DNAmAgeAcceleration: DNAmAgeAccel), and the main advantage of these biomarkers is the ability to quantify individual biological ageing. Therefore, targeting these DNAm ageing clocks and identifying which lifestyle habits decelerate or reverse the ageing processes would contribute to promoting healthy ageing.

At present, several potential “geroprotector” candidates (including rapamycin, sirtuin‐activating compounds, and metformin) are emerging in the field of ageing biology, and several clinical trials are being initiated based on the findings of model organisms (Kulkarni et al., 2022; Partridge et al., 2020); however, the validation of these pharmacological approaches remains insufficient. By contrast, exercise is a well‐documented effective “geroprotector” that reduces functional disability and age‐related diseases and extends health span (Chakravarty et al., 2008; Lear et al., 2017; Moore et al., 2012). Therefore, the relationships between physical activity and respective DNAmAgeAccel have been investigated. Specifically, some previous studies have reported a marginal negative relationship between physical activity and DNAmAgeAccel after adjusting for confounding factors (Fox et al., 2023; Kankaanpää et al., 2021; Kresovich et al., 2021; Quach et al., 2018; Spartano et al., 2023), while other papers have failed to find a relationship between them (Gale et al., 2018; Sillanpää et al., 2019, 2021) (Table S1). According to the results of the meta‐analysis, physical activity level is seemingly negatively associated with second‐generation clocks (i.e., DNAmPhenoAge and DNAmGrimAge) rather than with first‐generation clocks (i.e., DNAmAgeHorvath and DNAmAgeHannum) (Oblak et al., 2021). Previous studies assessed physical activity via self‐report questionnaires and accelerometers, and little is known about the relationship between the actual physical fitness, particularly VO_2max_, a strong predictor of all‐cause mortality, and DNAmAgeAccel (Jokai et al., 2023; Sillanpää et al., 2021). However, no study has directly calculated VO_2max_ from expiratory gas during the exercise stress test and investigated the relationship with DNAmAgeAccel in older people. Taken together, the relationship between physical activity and DNAmAgeAccel remains controversial, while that between actual physical fitness, including VO_2max_, and DNAmAgeAccel is largely unknown. It is essential to clarify the relationship between objectively measured actual physical fitness and DNAmAgeAccel, including direct measurements of VO_2max_, to develop a basis for establishing an efficient exercise intervention against biological ageing.

Regarding healthy lifestyle habits to slow down DNAmAgeAccel, lifestyle‐related variables other than physical fitness should be included naturally. In fact, lifestyle‐related variables such as body composition (Horvath et al., 2014; Kresovich et al., 2021; Levine et al., 2018; Lu et al., 2019; McCartney et al., 2018; Quach et al., 2018), diet (Levine et al., 2018; Lu et al., 2019; Quach et al., 2018), smoking (Kim et al., 2022; Levine et al., 2018; Lu et al., 2019), alcohol consumption (Kim et al., 2022; Quach et al., 2018), sleep status (Carroll et al., 2017; Carskadon et al., 2019; Freni‐Sterrantino et al., 2022), and blood biochemical parameters (Levine et al., 2018; Lu et al., 2019; Quach et al., 2018) are associated with the acceleration or deceleration of DNAm ageing clocks. However, to the best of our knowledge, the association of precise anthropometric parameters from magnetic resonance imaging (MRI) and dual‐energy X‐ray absorptiometry (DXA), nutrient intake status rather than blood nutritional parameters, and chronotype with DNAmAgeAccel is still unknown.

In addition, all DNAm ageing clocks were mainly developed by European, African, or Hispanic individuals, and studies using data from Asians are extremely limited (Lin, 2023). Previous studies have suggested that DNAm age differs among ethnic groups (Crimmins et al., 2021; Horvath et al., 2016; Tajuddin et al., 2019), but it remains unclear whether it reflects the same phenotype in Mongoloids as in other ethnic groups.

Therefore, this study primarily aims to determine the relationships between actual physical fitness, including peak oxygen uptake (VO_2peak_), and DNAmAgeAccel in older Japanese men. The secondary aim of this study is to determine the relative contribution to DNAmAgeAccel of lifestyle‐related variables, including precise anthropometric parameters, nutrient intake status, and chronotype. To achieve this purpose, we calculated five DNAm ageing clocks: DNAmAgeHorvath, DNAmAgeHannum, DNAmPhenoAge, DNAmGrimAge, and DNAmFitAge, and their AgeAccel using DNA samples from 144 Japanese men aged 65–72 years who participated in the Waseda Alumni's Sports, Exercise, Daily Activity, Sedentariness, and Health Study (WASEDA'S Health Study). Along with these five DNAm ageing clocks, we conducted various surveys and measurements, such as physical fitness, including direct VO_2max_ measurement by breath gas analysis, body composition, blood biochemical parameters, nutrients intake, smoking, alcohol consumption, disease status, sleep status, and chronotype. Through these surveys and measurements, we aim to identify lifestyle‐related variables, including physical fitness, associated with biological ageing and provide evidence for selecting targets for anti‐ageing intervention strategies.

## 2 RESULTS

### 2.1 Characteristics of the participants

The characteristics of all the participants are shown in Table 1 and Table S2. The overall number of participants in this study was 144 men. The mean chronological age was 68.0 ± 1.9 years, and all participants were Japanese.

**TABLE 1:** Characteristics of the participants.

	Mean ± SD			*n*
Anthropometric variables				
Height (cm)	168.0	±	5.8	144
Body weight (kg)	66.1	±	8.2	144
BMI (kg/m^2^)	23.4	±	2.5	144
Body fat (%)	21.2	±	5.4	144
Fat‐free mass (kg)	51.8	±	4.6	144
Visceral fat area (cm^2^)	96.5	±	45.6	139
Subcutaneous fat area (cm^2^)	123.6	±	48.3	139
Abdominal circumference (cm)	84.8	±	7.6	144
Calf circumference (cm)	36.6	±	2.1	136
Physical fitness variables and blood pressure				
VO_2_ at VT (mL/min)	1001.3	±	208.5	142
VO_2_/kg at VT (mL/kg/min)	15.3	±	3.4	142
VO_2_ at peak (mL/min)	1736.5	±	293.1	144
VO_2_/kg at peak (mL/kg/min)	26.5	±	4.6	144
Grip strength (kg)	35.2	±	5.7	143
Leg extension power (W)	1062.4	±	265.1	143
SBP (mmHg)	138.8	±	20.4	144
DBP (mmHg)	84.3	±	12.1	144
Blood biochemical variables				
Insulin (*μ*U/mL)	6.5	±	4.2	144
Glucose (mg/dL)	104.5	±	18.1	144
HbA1c (%)	5.7	±	0.5	144
TG (mg/dL)	111.7	±	62.6	144
Total‐C (mg/dL)	214.3	±	31.9	144
HDL‐C (mg/dL)	63.6	±	16.1	144
LDL‐C (mg/dL)	124.5	±	29.2	144
Nutrient variables				
Energy intake (kcal/day)	2106	±	558	144
Protein (g/1000 kcal/day)	42.0	±	7.4	144
Fat (g/1000 kcal/day)	32.0	±	6.9	144
CHO (g/1000 kcal/day)	117.8	±	21.9	144
Fe (mg/1000 kcal/day)	4.9	±	1.2	144
Zn (mg/1000 kcal/day)	4.6	±	0.7	144
Cu (mg/1000 kcal/day)	0.7	±	0.14	144
Mn (mg/1000 kcal/day)	1.6	±	0.4	144
Vitamin A (*μ*g RAE/1000 kcal/day)	516	±	268	144
Vitamin C (mg/1000 kcal/day)	74.2	±	31.2	144
*α*‐tocopherol (mg/1000 kcal/day)	4.6	±	1.1	144
*β*‐carotene (*μ*g/1000 kcal/day)	2335	±	1385	144
DNAmAge and DNAmAgeAccel				
Chronological age (years)	68.0	±	1.9	144
DNAmAgeHorvath (predicted years)	62.2	±	4.5	144
DNAmAgeHannum (predicted years)	55.1	±	3.8	144
DNAmPhenoAge (predicted years)	56.7	±	5.5	144
DNAmGrimAge (predicted years)	69.2	±	3.4	144
DNAmFitAge (predicted years)	71.7	±	3.5	144
HorvathAgeAccel (residuals)	0	±	4.4	144
HannumAgeAccel (residuals)	0	±	3.7	144
PhenoAgeAccel (residuals)	0	±	5.5	144
GrimAgeAccel (residuals)	0	±	3.2	144
FitAgeAccel (residuals)	0	±	3.3	144

*Note*: Data are mean ± standard deviation (SD).

Abbreviations: Accel, acceleration; BMI, body mass index; CHO, carbohydrate; Cu, copper; DBP, diastolic blood pressure; Fe, iron; HbA1c, Haemoglobin A1c; HDL‐C, high‐density lipoprotein cholesterol; LDL‐C, low‐density lipoprotein cholesterol; Mn, manganese; SBP, systolic blood pressure; TG, triglyceride; Total‐C, total cholesterol; VT, ventilatory threshold; Zn, zinc.

### 2.2 Chronological age, DNA methylation age, and DNA methylation age acceleration

**FIGURE 1:**
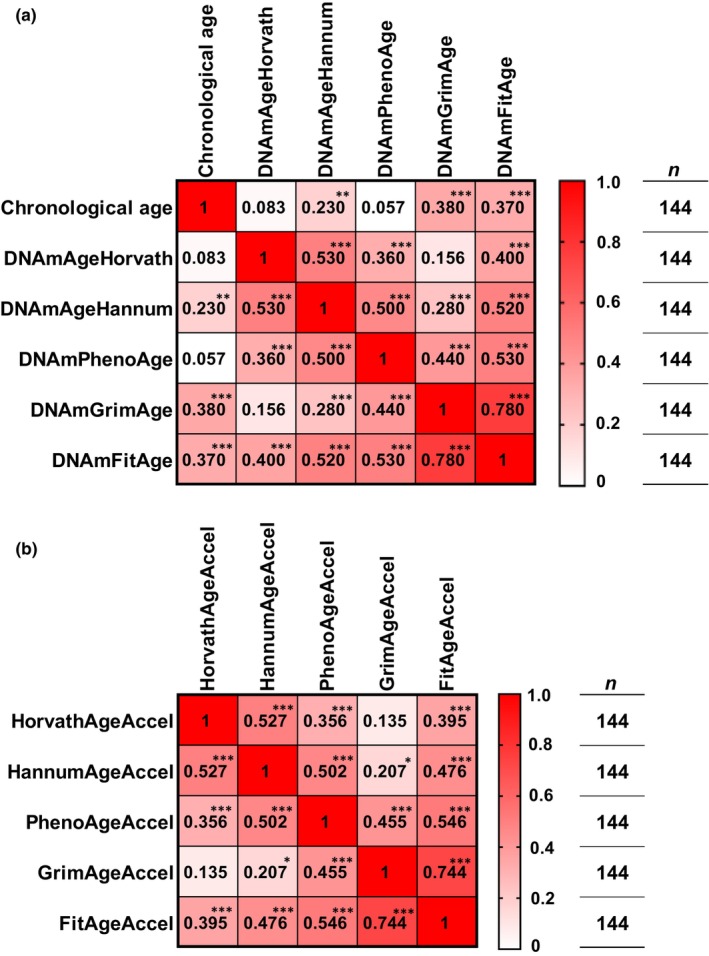
Correlations between chronological age, DNA methylation ageing clock, and DNA methylation age acceleration. (a) Correlations between chronological age and each DNA methylation ageing clock. (b) Correlations between each DNA methylation age acceleration. Significant correlations at *p* < 0.05, *p* < 0.01, and *p* < 0.001 are indicated by *, **, and ***, respectively.

The correlations between chronological age and DNAmAge are shown in Figure 1a. Chronological age significantly correlated with DNAmAgeHannum (*r* = 0.230, *p* < 0.01), DNAmGrimAge (*r* = 0.380, *p* < 0.001), and DNAmFitAge (*r* = 0.370, *p* < 0.001), while there was no significant correlation with DNAmAgeHorvath (*r* = 0.083, *p* = 0.321) and DNAmPhenoAge (*r* = 0.057, *p* = 0.501). The correlations between each DNAmAgeAccel are shown in Figure 1b.

### 2.3 Partial correlation analysis between DNAmAgeAccel with physical fitness and lifestyle‐related variables

**FIGURE 2:**
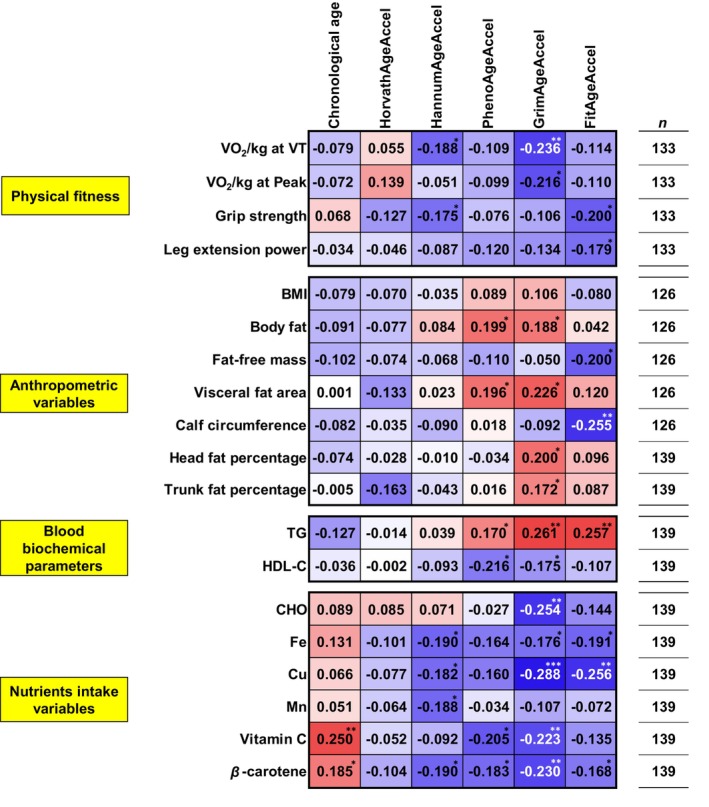
Partial correlation coefficients between lifestyle‐related variables and DNA methylation age acceleration. Data represent partial correlation coefficients (*r*) adjusted by chronological age, as well as smoking and drinking status (chronological age was adjusted by smoking and drinking status). Red cells indicate positive and blue cells indicate negative correlations. VO_2_, oxygen uptake; VT, ventilatory threshold; BMI, body mass index; TG, triglyceride; HDL‐C, high‐density lipoprotein cholesterol; CHO, carbohydrate; Fe, iron; Cu, copper; Mn, manganese. Significant correlations at *p* < 0.05, *p* < 0.01, and *p* < 0.001 are indicated by *, **, and ***, respectively.

The correlation heat maps between chronological age and DNAmAgeAccel and physical fitness including expiratory gas parameters, anthropometric variables, blood biochemical parameters, and nutrient intake are shown in Figures S1–S5. In summary, chronological age, HorvathAgeAccel, HannumAgeAccel, PhenoAgeAccel, GrimAgeAccel, and FitAgeAccel were significantly correlated with 11/137, 2/137, 12/137, 7/137, 19/137, and 7/137 variables, respectively. The correlation heat map between each DNAmAgeAccel and representative variables is shown in Figure 2.

In the physical fitness variables, VO_2_/kg at VT had a significant negative correlation with HannumAgeAccel (*r* = −0.188, *p* = 0.029) and GrimAgeAccel (*r* = −0.236, *p* = 0.006), and VO_2_/kg at Peak had a significant negative correlation with GrimAgeAccel (*r* = −0.216, *p* = 0.012). Grip strength had a significant negative correlation with HannumAgeAccel (*r* = −0.175, *p* = 0.042) and FitAgeAccel (*r* = −0.200, *p* = 0.020). Leg extension power had a significant negative correlation with FitAgeAccel (*r* = −0.179, *p* = 0.038). Chronological age had no significant correlation with any of the physical fitness variables.

In the anthropometric variables, both body fat and visceral fat area had significant positive correlations with PhenoAgeAccel (body fat: *r* = 0.199, *p* = 0.024; visceral fat area: *r* = 0.196, *p* = 0.027) and GrimAgeAccel (body fat: *r* = 0.188, *p* = 0.034; visceral fat area: *r* = 0.226, *p* = 0.010), respectively. Fat‐free mass and calf circumference had significant negative correlations with FitAgeAccel, respectively (fat‐free mass: *r* = −0.200, *p* = 0.024; calf circumference: *r* = 0.255, *p* = 0.004). However, body mass index (BMI) was not significantly correlated with DNAmAgeAccel. A more detailed analysis of body composition via DXA showed significant positive correlations between head and trunk fat percentages with GrimAgeAccel (head fat percentage: *r* = 0.200, *p* = 0.017; trunk fat percentage: *r* = 0.172, *p* = 0.042). Chronological age had no significant correlation with any of the anthropometric variables.

In the blood biochemical variables, triglyceride (TG) had significant positive correlations with PhenoAgeAccel (*r* = 0.170, *p* = 0.044), GrimAgeAccel (*r* = 0.261, *p* = 0.002), and FitAgeAccel (*r* = 0.257, *p* = 0.002). However, high‐density lipoprotein cholesterol (HDL‐C) had significant negative correlations with PhenoAgeAccel (*r* = −0.216, *p* = 0.010) and GrimAgeAccel (*r* = −0.175, *p* = 0.038). Chronological age had no significant correlation with both TG and HDL‐C.

In the nutrient intake variable, carbohydrate (CHO) had significant negative correlation with GrimAgeAccel (*r* = −0.254, *p* = 0.002). Both Fe and Cu had significant negative correlations with HannumAgeAccel (Fe: *r* = −0.190, *p* = 0.024; Cu: *r* = −0.182, *p* = 0.031), GrimAgeAccel (Fe: *r* = −0.176, *p* = 0.037; Cu: *r* = −0.288, *p* < 0.001), and FitAgeAccel (Fe: *r* = −0.191, *p* = 0.024; Cu: *r* = −0.256, *p* = 0.002). Mn had a significant negative correlation only with HannumAgeAccel (*r* = −0.188, *p* = 0.025). Vitamin C had significant negative correlations with PhenoAgeAccel (*r* = −0.205, *p* = 0.015) and GrimAgeAccel (*r* = −0.223, *p* = 0.008). *β*‐carotene had significant negative correlations with HannumAgeAccel (*r* = −0.190, *p* = 0.024), PhenoAgeAccel (*r* = −0.183, *p* = 0.030), GrimAgeAccel (*r* = −0.230, *p* = 0.006), and FitAgeAccel (*r* = −0.168, *p* = 0.046). Vitamin C and *β*‐carotene had a significant positive correlation with chronological age (vitamin C: *r* = 0.250, *p* = 0.003; *β*‐carotene: *r* = 0.185, *p* = 0.027).

### 2.4 DNA methylation age acceleration for each continuous variable via analysis of covariance

**FIGURE 3:**
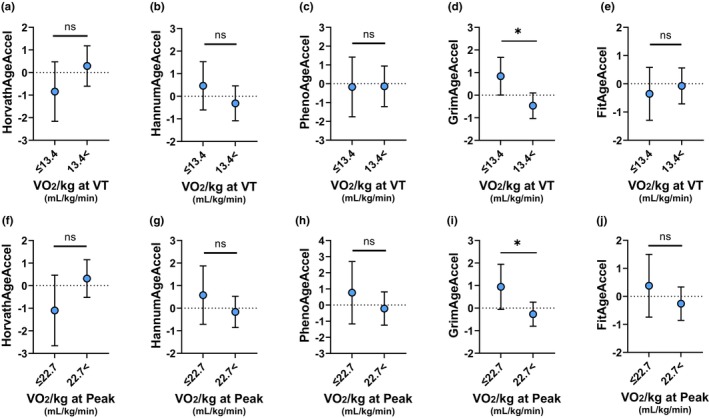
DNA methylation age acceleration of cardiorespiratory fitness by analysis of covariance. (a–e) Comparison of each DNA methylation age acceleration in the two groups of oxygen uptake per kg at ventilatory threshold (VO_2_/kg at VT). (f–j) Comparison of each DNA methylation age acceleration in the two groups of oxygen uptake per kg at peak (VO_2_/kg at Peak). Data represent means ±95% confidence intervals adjusted by chronological age, as well as smoking and drinking status. Significant correlations at *p* < 0.05 is indicated by *.

The results of the analysis of covariance (ANCOVA) for cardiorespiratory fitness (CRF) are shown in Figure 3 and Table S3. On the one hand, in both VO_2_/kg at VT and VO_2_/kg at Peak, GrimAgeAccel was significantly lower in the higher fitness group than in the lower fitness group (VO_2_/kg at VT: *p* = 0.011; VO_2_/kg at Peak: *p* = 0.037) (Figure 3d,i). On the contrary, there were no significant differences between groups in DNAmAgeAccel (Figure 3a–c,e–h,j).

**FIGURE 4:**
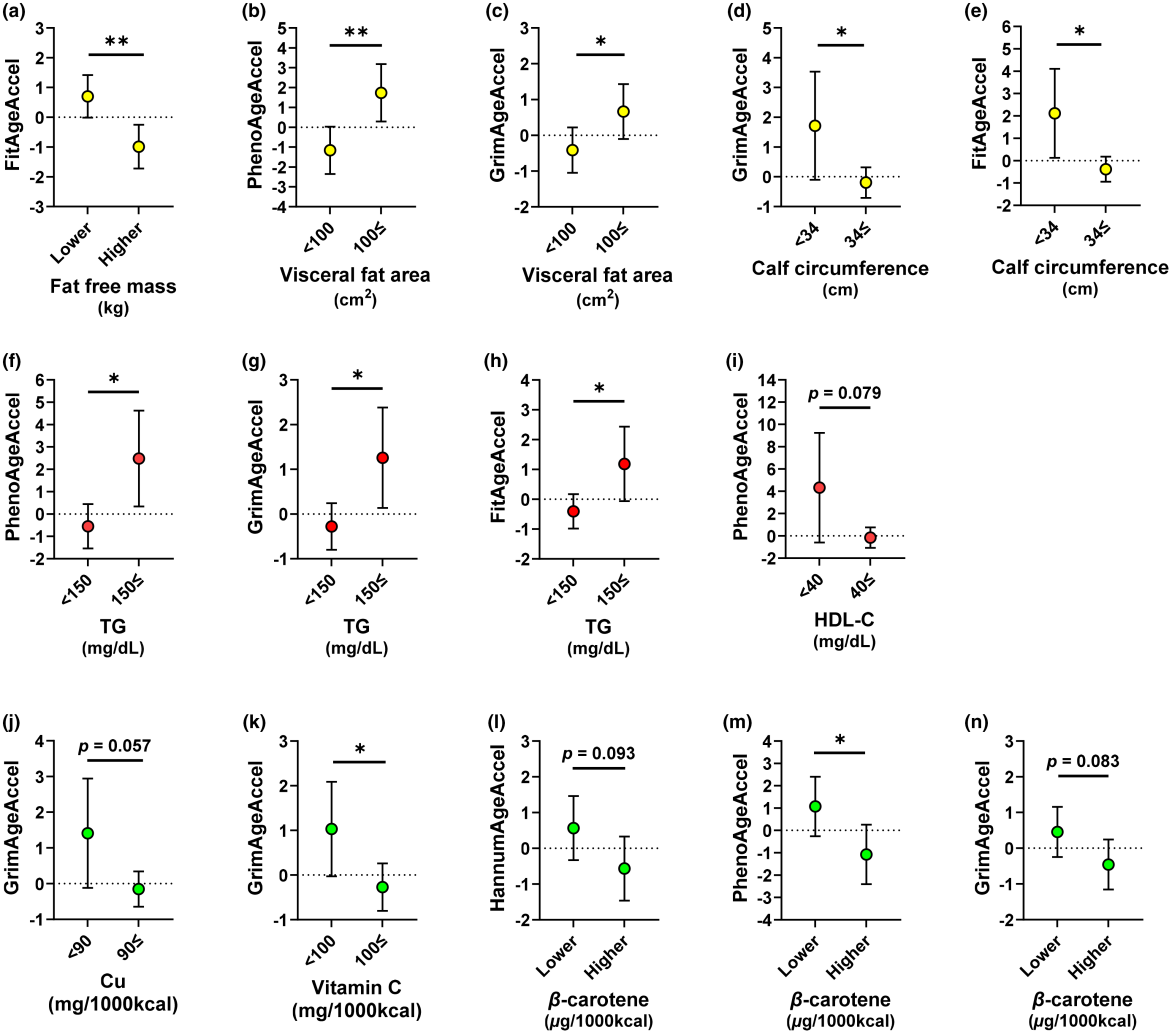
DNA methylation age acceleration of lifestyle‐related variables by analysis of covariance. (a) Comparison of FitAgeAcceleration in the two groups of fat‐free mass. (b, c) Comparison of PhenoAgeAcceleration and GrimAgeAcceleration in the two groups of visceral fat area. (d, e) Comparison of GrimAgeAcceleration and FitAgeAcceleration in the two groups of calf circumference. (f–h) Comparison of PhenoAgeAcceleration, GrimAgeAcceleration, and FitAgeAcceleration in the two groups of triglyceride (TG). (i) Comparison of PhenoAgeAcceleration in the two groups of high‐density lipoprotein cholesterol (HDL‐C). (j) Comparison of GrimAgeAcceleration in the two groups of copper (Cu). (k) Comparison of GrimAgeAcceleration in the two groups of vitamin C. (l–n) Comparison of HannumAgeAcceleration, PhenoAgeAcceleration, and GrimAgeAcceleration in the two groups of *β*‐carotene. Data represent means ±95% confidence intervals adjusted by chronological age, as well as smoking and drinking status. Significant correlations at *p* < 0.05 and *p* < 0.01 are indicated by * and **, respectively.

The results of the ANCOVA for other lifestyle‐related variables are shown in Figure 4 and Table S3. In the anthropometric variables, FitAgeAccel was significantly lower in the higher fat‐free mass group than in the lower fat‐free mass group (*p* = 0.002) (Figure 4a). Clinical cutoff results showed that PhenoAgeAccel and GrimAgeAccel were significantly higher in the higher visceral fat area group than in the lower visceral fat area group (PhenoAgeAccel: *p* = 0.003; GrimAgeAccel: *p* = 0.037) (Figure 4b,c). GrimAgeAccel and FitAgeAccel were significantly lower in the higher calf circumference group than in the lower calf circumference group (GrimAgeAccel: *p* = 0.048; FitAgeAccel: *p* = 0.018) (Figure 4d,e).

In the blood biochemical variables, PhenoAgeAccel, GrimAgeAccel, and FitAgeAccel were significantly higher in the higher TG group than in the lower TG group (PhenoAgeAccel: *p* = 0.013; GrimAgeAccel: *p* = 0.016; FitAgeAccel: *p* = 0.025) (Figure 4f–4h). However, PhenoAgeAccel tended to be lower in the higher HDL‐C group than in the lower HDL‐C group (*p* = 0.079) (Figure 4i **)**.

In the nutrient intake variables, GrimAgeAccel was significantly lower in the higher vitamin C intake group than in the lower vitamin C intake group (*p* = 0.034) (Figure 4k **)**. PhenoAgeAccel was significantly lower in the higher *β*‐carotene intake group than in the lower *β*‐carotene intake group (*p* = 0.032) (Figure 4m). HannumAgeAccel (*p* = 0.093) and GrimAgeAccel (*p* = 0.083) tended to be lower in the higher *β*‐carotene intake group than in the lower *β*‐carotene intake group (Figure 4l,n **)**. GrimAgeAccel tended to be lower in the higher Cu intake group than in the lower Cu intake group (*p* = 0.057) (Figure 4j **)**.

### 2.5 DNA methylation age acceleration of the categorical variables

**FIGURE 5:**
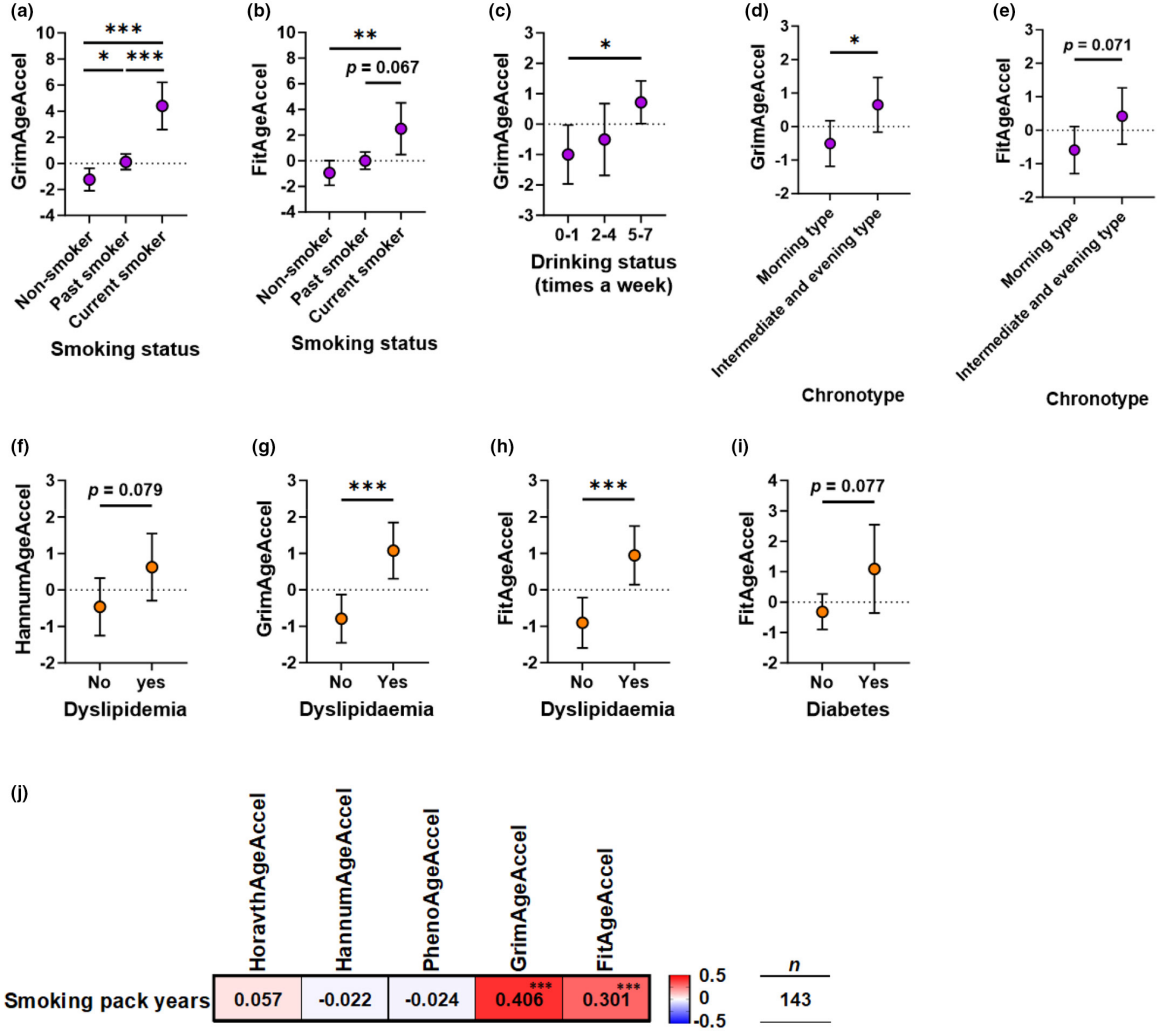
DNA methylation age acceleration of categorical variables by analysis of covariance. (a, b) Comparison of GrimAgeAcceleration and FitAgeAcceleration in the three groups of smoking status. (c) Comparison of GrimAgeAcceleration in the three groups of drinking status. (d, e) Comparison of GrimAgeAcceleration and FitAgeAcceleration in the two groups of chronotype. (f–h) Comparison of HannumAgeAcceleration, GrimAgeAcceleration, and FitAgeAcceleration in the two groups of hyperlipidaemia. (i) Comparison of FitAgeAcceleration in the two groups of diabetes. (j) Partial correlation coefficient between smoking pack years and each of DNA methylation age acceleration. Data represent means ±95% confidence intervals or partial correlation coefficients (*r*) adjusted by chronological age. Red cells indicate positive and blue cells indicate negative correlations. Significant differences or correlations at *p* < 0.05, *p* < 0.01, and *p* < 0.001 are indicated by *, ** and ***, respectively.

The results of the ANCOVA for the categorical variables are shown in Figure 5 and Tables S4 and S5. Regarding smoking status, current smokers had higher GrimAgeAccel (*p* < 0.001) and FitAgeAccel (*p* = 0.008) than non‐smokers (Figure 5a,b, and Table S4). The GrimAgeAccel of past smokers was also higher than that of non‐smokers (*p* = 0.035). Furthermore, the GrimAgeAccel of current smokers was higher than that of past smokers (*p* < 0.001). Correlation analysis of smoking pack years with each DNAmAgeAccel showed a significant correlation between the smoking pack years and the GrimAgeAccel (*r* = 0.406, *p* < 0.001) and FitAgeAccel (*r* = 0.301, *p* < 0.001), respectively (Figure 5j). For drinking status, the group that drank 5–7 times a week had a higher GrimAgeAccel than the group that drank once a week or less (*p* = 0.016) (Figure 5c and Table S4). For the Pittsburgh Sleep Quality Index (PSQI) score, there was no difference in any of the DNAmAgeAccel constructs by the presence or absence of sleep disorders (Table S4). For the morningness–eveningness questionnaire (MEQ) score, the GrimAgeAccel of the moderate and definite morning type group was significantly lower than the intermediate and moderate evening type group (*p* = 0.033), and there was a significant trend for FitAgeAccel (*p* = 0.071) (Figure 4d,e, and Table S4). For each disease, GrimAgeAccel and FitAgeAccel were significantly higher in the group with dyslipidemia than those in the group without the disease (*p* < 0.001), and there was a significant trend in HannumAgeAccel (*p* = 0.079) (Figure 4f–h and Table S5). There was a significant trend for FitAgeAccel in the group with and without diabetes (Figure 4i and Table S5).

### 2.6 Multiple regression analysis of each DNA methylation age acceleration

The results of the multiple regression analysis using the forced entry method for each DNAmAgeAccel are presented in Table 2. A significant regression equation was obtained for GrimAgeAccel (*p* < 0.001) and FitAgeAccel (*p* < 0.001), although it was not significant for PhenoAgeAccel (*p* = 0.139). The contribution of the 16 variables entered (adjusted R^2^) was 5.6% for PhenoAgeAccel, 32.9% for GrimAgeAccel, and 21.4% for FitAgeAccel.

**TABLE 2:** Multiple regression analysis of each DNA methylation age acceleration.

	β	95% CI	Standard *β*	VIF	*p*
PhenoAgeAccel^a^	―	―	―	―	―
GrimAgeAccel^b^					
Intercept	0.304	−25.890 to 26.499			0.982
VO_2_/kg at VT (mL/kg/min)	−0.172	−0.406 to 0.062	−0.185	3.041	0.148
VO_2_/kg at Peak (mL/kg/min)	0.064	−0.127 to 0.255	0.094	3.755	0.508
Grip strength (kg)	0.001	−0.080 to 0.082	0.002	1.062	0.981
Fat‐free mass (kg)	−0.043	−0.190 to 0.104	−0.062	2.118	0.563
Visceral fat area (cm^2^)	0.002	−0.011 to 0.015	0.030	1.836	0.766
Calf circumference (cm)	−0.298	−0.608 to 0.011	−0.196	1.977	0.059
Head fat (%)	0.814	−0.341 to 1.97	0.121	1.403	0.165
TG (mg/dL)	0.014	0.006 to 0.023	0.286	1.400	0.001**
HDL‐C (mg/dL)	−0.003	−0.040 to 0.035	−0.013	1.758	0.890
CHO (g/1000 kcal)	−0.036	−0.061 to −0.011	−0.258	1.505	0.005**
Cu (mg/1000 kcal)	−2.402	−7.397 to 2.594	−0.102	2.172	0.343
Vitamin C (mg/1000 kcal)	0.004	−0.021 to 0.030	0.042	3.101	0.744
*β*‐carotene (*μ*g/1000 kcal)	−0.0003	−0.001 to 0.00033	−0.129	3.585	0.352
Smoking	1.495	0.575 to 2.416	0.266	1.286	0.002**
Drinking	−0.032	−0.669 to 0.606	−0.009	1.456	0.921
Chronotype	−0.579	−1.600 to 0.442	−0.090	1.191	0.263
FitAgeAccel^c^					
Intercept	13.545	−15.824 to 42.914			0.363
VO_2_/kg at VT (mL/kg/min)	−0.043	−0.306 to 0.220	−0.045	3.041	0.747
VO_2_/kg at Peak (mL/kg/min)	0.049	−0.165 to 0.262	0.069	3.755	0.653
Grip strength (kg)	−0.067	−0.158 to 0.024	−0.119	1.062	0.148
Fat‐free mass (kg)	−0.048	−0.212 to 0.117	−0.066	2.118	0.565
Visceral fat area (cm^2^)	0.003	−0.012 to 0.018	0.046	1.836	0.668
Calf circumference (cm)	−0.433	−0.780 to −0.085	−0.274	1.977	0.015*
Head fat (%)	0.433	−0.863 to 1.729	0.062	1.403	0.509
TG (mg/dL)	0.018	0.008 to 0.027	0.341	1.400	< 0.001***
HDL‐C (mg/dL)	0.008	−0.034 to 0.049	0.038	1.758	0.717
CHO (g/1000 kcal)	−0.027	−0.055 to 0.001	−0.185	1.505	0.058
Cu (mg/1000 kcal)	−4.073	−9.673 to 1.528	−0.168	2.172	0.152
Vitamin C (mg/1000 kcal)	0.014	−0.014 to 0.043	0.139	3.101	0.321
*β*‐carotene (*μ*g/1000 kcal)	−0.0003	−0.001 to 0.0004	−0.144	3.585	0.338
Smoking	0.935	−0.097 to 1.967	0.161	1.286	0.075
Drinking	−0.237	−0.952 to 0.478	−0.063	1.456	0.512
Chronotype	−0.584	−1.728 to 0.561	−0.087	1.191	0.314

Abbreviations: CHO, carbohydrate; CI, confidence intervals; Cu, copper; GrimAgeAccel, GrimAgeAcceleration; HDL‐C, high‐density lipoprotein cholesterol; PhenoAgeAccel, PhenoAgeAcceleration; TG, triglyceride; VIF, variance inflation factor; VO_2_, oxygen uptake; VT, ventilatory threshold. Significant differences at *p* < 0.05, *p* < 0.01 and *p* < 0.001 are indicated by *, ** and ***, respectively.

^a^ *R* = 0.430, adjusted *R* ^2^ = 0.056.

^b^ *R* = 0.648, adjusted *R* ^2^ = 0.329.

^c^ *R* = 0.567, adjusted *R* ^2^ = 0.214.

## 3 DISCUSSION

Novel findings of this study include that the maintenance of high CRF, abundant intake of CHO and antioxidants, and morning type chronotype are associated with delayed biological ageing in older men. We also find that higher fat‐free mass and calf circumference and lower visceral fat area, rather than BMI, are associated with delayed biological ageing. Multiple regression analysis suggested that calf circumference, serum TG, CHO intake, and smoking status, rather than CRF, contribute more to biological ageing.

Most of the previous studies on the relationship between exercise and biological ageing, as measured by DNAm ageing clocks, have been conducted on the basis of self‐reported questionnaires and accelerometers (Fox et al., 2023; Gale et al., 2018; Kankaanpää et al., 2021; Kresovich et al., 2021; Quach et al., 2017; Sillanpää et al., 2019, 2021; Spartano et al., 2023). These previous studies have concluded that there is either a weak negative relationship or no relationship between physical activity and DNAmAgeAccel as adjusted by confounders. These findings suggest that moderate physical activity is at least partially involved in delaying biological ageing. However, rigorously defined, physical activity and physical fitness are different concepts, and physical fitness is the result of exercise, which is planned and regular physical activity (Caspersen et al., 1985). In particular, VO_2max_ is the most representative indicator of physical fitness with a reported relationship to all‐cause mortality (Blair et al., 1989). Our cohort study has the advantage of performing exercise stress tests in older adults, which allows us to analyse detailed expiratory gas parameters. The present study provides new insights into the relationship between detailed expiratory gas parameters and biological ageing using an exercise physiology approach, in addition to the epidemiologic approach to large‐scale physical activity survey that have been conducted to date. More specifically, we found that VO_2_/kg at VT and VO_2_/kg at Peak were negatively correlated with GrimAgeAccel, even after adjustment by cofounders such as chronological age, as well as smoking and drinking status.

Besides physical activity, previous studies have reported a negative relationship between FEV1, which represents lung function, and DNAmAgeAccel (Maddock et al., 2020; Marioni et al., 2015). FEV1 has also been shown to correlate positively with VO_2max_ (Rasch‐Halvorsen et al., 2019), and thus, the negative relationship between VO_2peak_ and GrimAgeAccel observed in this study can be interpreted as a reasonable result. From a physiological perspective, however, VO_2max_ is assessed by gas exchange capacity in the lungs, cardiac output, oxygen transport capacity in blood, and oxygen uptake capacity in skeletal muscle, and lung function is only one of the limiting factors of VO_2max_ (Bassett & Howley, 2000). In other words, VO_2max_ reflects more multiorgan and systemic functions compared to FEV1. In addition, the improvement of VO_2max_ caused by exercise training is essentially determined by the relatively preserved oxygen‐carrying capacity and the increase in stroke volume (Lundby et al., 2017). Therefore, investigating the relationship between biological ageing and VO_2max_ would provide physiological data that are fundamentally different from FEV1. To our knowledge, previous studies that have investigated the relationship between VO_2max_ and DNAmAgeAccel have used indirectly calculated VO_2max_ values or measured DNAmAgeAccel using skeletal muscle in young adults (Jokai et al., 2023; Sillanpää et al., 2021). The present study measured VO_2peak_ directly by expiratory gas analysis in older men and found that GrimAgeAccel was significantly higher in the group with VO_2_/kg at VT and VO_2_/kg at Peak below reference values. This result indicates that maintaining high CRF may contribute to delaying biological ageing, from another perspective, the results may suggest that excessive reduction in CRF accelerates DNAmAgeAccel. This is consistent with the concept in exercise science that states that even a minimum amount of physical activity can provide health‐promoting effects (Arem et al., 2015; Wen et al., 2011). In fact, a recent report indicates that the health benefits on biological ageing of additional physical activity are likely to be greatest in adults who lead sedentary lifestyles (Fox et al., 2023). In addition, as VT levels are strongly influenced by the oxygen uptake capacity in skeletal muscle (Bassett & Howley, 2000), the negative relationship between VO_2_/kg at VT and GrimAgeAccel in this study may suggest that the high metabolic capacity of skeletal muscle is associated with delayed biological ageing.

Most previous studies investigating the relationships between anthropometric variables and DNAm ageing clocks have used BMI as an indicator, and some systematic reviews have reported a positive relationship between them (Oblak et al., 2021; Ryan et al., 2020). However, in this study, body fat percentage, fat‐free mass, and calf circumference, rather than BMI, were most associated with PhenoAgeAccel, GrimAgeAccel, and FitAgeAccel. Furthermore, detailed analysis using MRI and DXA revealed that among whole body parts, trunk fat percentage, especially visceral fat accumulation, was most associated with accelerated PhenoAgeAccel and GrimAgeAccel. These trends were similar in the classification of subjects using clinical cutoffs. Although our results are consistent with those of previous studies showing that visceral fat accumulation is positively correlated with GrimAgeAccel (Arpón et al., 2019; Lu et al., 2019), this is the first study to demonstrate that the maintenance of fat‐free mass and calf circumference are negatively correlated with FitAgeAccel. As fat‐free mass is primarily determined by skeletal muscle mass, the negative correlation between muscle strength (i.e., grip strength) and muscle power (i.e., leg extension power) and FitAgeAccel observed in this study are plausible results. However, the differences in results with previous studies observed in BMI could be attributed to the relatively small range of BMI (20.9–26.2) of the participants in this study. However, the findings of this study indicate that visceral fat area, fat‐free mass, and calf circumference can be important variables for estimating biological ageing, even in older men with relatively homogeneous BMIs.

In other continuous variables, the blood biochemical parameters TG and HDL‐C were previously shown to be positively or negatively correlated with PhenoAgeAccel, GrimAgeAccel, and FitAgeAccel (Jokai et al., 2023; Levine et al., 2018; Lu et al., 2019). These blood parameters are also known to correlate well with physical fitness levels and body composition, suggesting the importance of maintaining an optimal metabolic energy state for delaying biological ageing. Notably, HDL‐C is widely used in clinical settings, mainly as a biomarker for estimating cardiovascular risk. However, a recent study suggests that there is a J‐shaped dose–response manner between blood HDL‐C levels and cardiovascular diseases and all‐cause mortality (Zhong et al., 2020). Therefore, the upper limit of the blood HDL‐C level that has a favourable effect on biological ageing is currently unknown. In the nutrient intake variables, limited evidence suggests that nutritional biomarkers in blood, such as *β*‐carotene and carotenoids, are negatively associated with PhenoAgeAccel and GrimAgeAccel (Levine et al., 2018; Lu et al., 2019). However, the relationship between DNAmAgeAccel and daily intake of nutrients largely remains unknown. Our findings reveal that the adequate intake of CHO and micronutrients are associated with delayed biological age progression.

In the categorical variables, it should be emphasized that morning chronotype was associated with delayed GrimAgeAccel. Some studies have examined the relationship between chronotype and DNA methylation patterns (Wong et al., 2015), but to our knowledge, no study has investigated the relationship with the DNAmAgeAccel. In other variables, the positive relationships between smoking, excessive alcohol consumption, and dyslipidemia with GrimAgeAccel and FitAgeAccel are roughly consistent with the findings of previous studies (Freni‐Sterrantino et al., 2022; Kim et al., 2022; Oblak et al., 2021). However, categorical variables, such as the presence of sleep disorders, diabetes, hypertension, and lifestyle‐related diseases, showed either no or marginal relationship with DNAmAgeAccel. Taken together, these results suggest that maintaining a morning life‐rhythm and an adequate energy balance, as well as avoidance of smoking and excessive alcohol consumption, are associated with delayed biological ageing.

Partial correlation analysis and ANCOVA results indicated that GrimAgeAccel and FitAgeAccel may be more suited to capture individual phenotypes than chronological age in relatively homogeneous subjects such as age, gender, and race. In addition, the results suggest that these two clocks reflect lifestyle‐related variables better than other DNAm ageing clocks and can capture similar phenotypes even for different races. In fact, in multiple regression analysis, significant regression models were obtained only for GrimAgeAccel and FitAgeAccel. The standardised partial regression coefficients were larger for TG, smoking, and CHO in GrimAgeAccel, and for TG and calf circumference in FitAgeAccel, in this order. The negative relationship between GrimAgeAccel and smoking is well known, and the relationship between GrimAgeAccel and TG and CHO was also reported in larger sample sizes than in this study when this clock was developed (Lu et al., 2019). Therefore, the new finding of this study would be a negative relationship between FitAgeAccel and calf circumference. On the other hand, the relative contribution of VO_2_/kg at VT and VO_2_/kg at Peak to these two clocks appear to be low. However, as daily exercise is closely related to other lifestyle‐related variables such as energy metabolism, body composition, nutrient intake, and chronotype, the results of multiple regression analysis would not imply that the maintenance of CRF is not beneficial for biological ageing.

In the present study, we demonstrated only associations between lifestyle‐related variables, including CRF, and DNAm ageing clocks, and the causal relationship between them remains unclear. In fact, DNAm ageing clocks are strongly influenced by the datasets as they are developed, thereby suggesting that each clock reflects a different aspect of biological ageing (Dabrowski et al., 2023; Zhang et al., 2019). The present study mainly showed the relationship between DNAmGrimAge, DNAmFitAge developed based on DNAmGrimAge, and lifestyle‐related factors including CRF. However, future longitudinal studies should investigate the relationships between changes in lifestyle‐related variables, including CRF, with DNAmAgeAccel. At present, the number of human intervention studies targeting DNAm ageing clocks is limited (Fahy et al., 2019; Fiorito et al., 2021; Fitzgerald et al., 2021; Waziry et al., 2023). Importantly, these studies suggest that long‐term pharmacological or lifestyle interventions are effective in not only slowing down but also reversing DNAmAgeAccel. In addition, it should be mentioned that exercise promotes systemic health‐promoting effects through the physiological effects of “exerkines” (Chow et al., 2022). Put differently, the delayed and reversed effects of exercise on DNAmAgeAccel may be systemic and organ‐specific. Therefore, it is also necessary to determine whether cardiometabolic improvements induced by exercise interventions are effective in slowing or reversing DNAmAgeAccel in multiple organs, not only in the blood. Furthermore, racial and sex differences in DNAmAgeAccel should be considered (Horvath et al., 2016; Tajuddin et al., 2019).

## 4 CONCLUSIONS

In conclusion, the contribution of CRF to GrimAgeAccel and FitAgeAccel is relatively low compared to lifestyle‐related factors such as serum TG, daily intake of CHO, smoking, and calf circumference. However, this study reveals a negative correlation between CRF with GrimAgeAccel, even after adjustment by chronological age, as well as smoking and drinking status, and the aforementioned reference values are negatively associated with delayed GrimAgeAccel. These results indicate that maintaining high CRF may contribute to delaying biological ageing.

## 5 METHODS

### 5.1 Participants

The WASEDA'S Health Study is a prospective cohort study that investigates the relationships between health outcomes and sports, exercise, physical activity, and sedentary behaviour among the alumni of Waseda University and their spouses aged 40 years or older (Ito et al., 2019; Ito, Tanisawa, et al., 2019; Kawamura et al., 2021; Tanisawa et al., 2022a, 2022b). The WASEDA'S Health Study consists of four cohorts (A–D) with different measurement items, and the participants selected one of the four cohorts when registering for the study (Tanisawa et al., 2022b). The study included a total of 169 men aged 65–72 years who participated in the baseline survey of Cohort D between March 2015 and March 2020, 144 of whom were included in order of measurement date from the earliest to the latest, after excluding those whose DNA sample quality did not meet the criteria (*n* = 11). The participants were briefed on the study and signed an informed consent form prior to the baseline survey. This study was approved by the Research Ethics Committee of Waseda University (approval numbers: 2014‐G002 and 2018‐G001). The study was conducted in accordance with the Declaration of Helsinki (1964).

### 5.2 Self‐administered questionnaires and nutrient assessment

Self‐administered questionnaires were used to determine the following: age (in years), smoking habits (current, former, and non‐smoking), and frequency of drinking (less than once a week, 2–4 times a week, and more than 5 times a week). Sleep quality was assessed using the PSQI score (Backhaus et al., 2002; Doi et al., 2000), with a score of 5 points or less defined as no sleep disorder and 6 points or more as having a sleep disorder. Chronotype was assessed using the Japanese version of the MEQ (Ishihara et al., 1986; Ostberg & Horne, 1976). The MEQ consisted of 19 questions about preferred sleep time and daily performance, and the scores ranged from 16 to 86. Based on the MEQ score, the participants were classified into intermediate and moderate evening type (score 31–58) or morning and definite morning type (59–86). As described previously (Ito et al., 2019; Ito, Tanisawa, et al., 2019), the brief‐type self‐administered dietary history questionnaire (BDHQ) was also used to investigate the intake of total energy (kcal/day), protein (g/1000 kcal/day), fat (g/1000 kcal/day), CHO (g/1000 kcal/day), iron (Fe, mg/1000 kcal/day), zinc (Zn, mg/1000 kcal/day), copper (Cu, mg/1000 kcal/day), manganese (Mn, mg/1000 kcal/day), vitamin A (calculated as retinol activity equivalent, *μ*gRAE/1000 kcal/day), vitamin C (mg/1000 kcal/day), α‐tocopherol (mg/1000 kcal/day), and *β*‐carotene (calculated as *β*‐carotene equivalent, *μ*g/1000 kcal/day). The validity of the BDHQ has been evaluated in previous studies (Kobayashi et al., 2011, 2012).

### 5.3 Anthropometric measurements

Height (cm) and body weight (kg) were measured using a stadiometer (YHS‐200D, YAGAMI Inc., Nagoya, Japan) and an anthropometer (MC‐980A, Tanita, Tokyo, Japan), respectively. Body weight was measured with light clothing and without shoes. BMI (kg/m^2^) was calculated from height and body weight measurements. Body fat (%) was measured using bioelectrical impedance analysis (MC‐980A, Tanita, Tokyo, Japan). Fat‐free mass (kg) was calculated from the body weight and fat. Visceral fat area (cm^2^) and subcutaneous fat area (cm^2^) were measured using MRI (Signa Premier; GE Healthcare, Waukesha, WI, USA), as described previously (Usui et al., 2020). Body composition at each body part was measured using DXA, as described in a previous study (Kawakami et al., 2021). Abdominal circumference (cm) was measured to the nearest 0.1 cm at the umbilical region with an inelastic measuring tape at the end of normal expiration. Calf circumference (cm) was measured in 0.1 cm increments in the standing position; twice on each side where the circumference was the greatest. Details of the two circumferential measurements can be found in previous studies (Kawakami et al., 2020).

### 5.4 Blood pressure and physical fitness measurements

Resting systolic and diastolic blood pressures (SBP and DBP, mmHg) were measured at least twice in the sitting position using an automatic sphygmomanometer (HEM‐7250‐IT; Omron Healthcare Co., Ltd., Kyoto, Japan), and the mean value was used (Tanisawa et al., 2022b). VO_2peak_ (mL/min and mL/kg/min), grip strength (kg), and leg extension power (W) were measured as previously reported (Kawamura et al., 2021). Specifically, VO_2peak_ was measured using the breath‐by‐breath method with a bicycle ergometer (828E, Monarch, Stockholm, Sweden). Measurements started at 30 W after 3 min of rest, and the load was gradually increased by 15 W/min until exhaustion. The endpoint of the exercise stress test was defined as the plateau of oxygen uptake, or the heart rate reached approximately 90% of the predicted maximum age‐specific heart rate. The exercise test was stopped if the perceived exertion's rating reached 18 or higher, if the subject reported that he could not continue exercising, or if the SBP reached 250 mmHg. VO_2peak_ was defined as the highest value of average oxygen uptake during 30 s of exercise, and other expiratory gas‐related parameters, including VO_2_ at VT (mL/min and mL/kg/min) were also calculated. Heart rate (bpm) and blood pressure were monitored before and during exercise for the safety of the older participants. Grip strength was measured twice, alternately on the left and right hand, and the average value for the right hand was used in this study. Leg extension power was repeatedly measured five times using a recumbent leg press (Anaero Press 3500, Combi, Tokyo, Japan) and the maximum value was adopted.

### 5.5 Blood sampling and biochemical measurements

The procedure from blood collection to blood sample storage was as previously reported (Kawamura et al., 2021). The participants were briefly instructed to fast for at least 12 h on the night before the blood collection. Venous blood was collected from a forearm vein into blood collection tubes (TERUMO, Tokyo, Japan) with or without anticoagulant (EDTA‐2Na with NaF and heparin sodium) and centrifuged at 4°C for 10 min at 3000 rpm using a centrifuge (Model 5911, Kubota, Tokyo, Japan). The supernatant was transferred to microtubes, and both plasma and serum samples were stored in a −80°C freezer until analysis. The serum samples were analysed for insulin (*μ*U/mL), TG (mg/dL), total cholesterol (total‐C, mg/dL), HDL‐C (mg/dL), and low‐density lipoprotein cholesterol (LDL‐C, mg/dL). The plasma samples were analysed for fasting blood glucose (glucose, mg/dL) and haemoglobin A1c (HbA1c, %). All analyses of these blood biochemical parameters were performed by BML, Inc. (Tokyo, Japan).

### 5.6 Definition of diseases

According to the guidelines of the Japan Atherosclerosis Society (Teramoto et al., 2012), dyslipidaemia is defined as having at least one of the following components: fasting LDL levels ≥140 mg/dL, fasting HDL‐C levels <40 mg/dL, and fasting TG levels ≥150 mg/dL. Additionally, according to the guidelines of the Japanese Society of Hypertension's guidelines (Umemura et al., 2019), hypertension is defined as having both or either of the following components: SBP ≥140 mmHg and DBP ≥90 mmHg. According to the guidelines of the Japan Diabetes Society (Araki et al., 2020), diabetes is defined as having both or either fasting glucose ≥126 mg/dL and HbA1c ≥6.5%. Lifestyle‐related diseases are defined as having one or more of the above, dyslipidaemia, hypertension, or diabetes mellitus.

### 5.7 DNA extraction and epigenome‐wide DNA methylation measurement

For DNA extraction, blood was drawn into collection tubes containing anticoagulant (EDTA‐2Na). DNA extraction using whole blood was performed according to the manufacturer's instructions using QIAamp DNA Midi Kit (Qiagen, Germany). The extracted DNA was dissolved in Buffer AE (10 mM Tris‐Cl, 0.5 mM EDTA, pH 9.0) Prior to DNA methylation measurement, DNA samples were adjusted to a concentration of ≥50 ng/μL with a purity of A260/280 ranging from >1.7 to 2.1.

Epigenome‐wide DNA methylation was measured using the same procedure as previously reported (Jokai et al., 2023). Briefly, genomic DNA bisulfite conversion was performed by using the EZ DNA Methylation Kit (Zymo Research, Irvine, CA, USA), followed by hybridisation using Infinium MethylationEPIC BeadChip Kit (Illumina Inc., San Diego, CA, USA). Sample‐ and probe‐based quality checking were performed using R version 4.0.5, and they involved the use of minfi, Meffil, and ewastools R packages (Jokai et al., 2023). In this study, no samples were excluded, as all 144 DNA samples passed the criteria set by Illumina.

### 5.8 Epigenetic biomarkers

Five DNAm ageing clocks—DNAmAgeHorvath (years), DNAmAgeHannum (years), DNAmPhenoAge (years), DNAmGrimAge (years), and DNAmFitAge (years)—were evaluated using the obtained epigenome‐wide DNA methylation data as previously reported (Hannum et al., 2013; Horvath, 2013; Levine et al., 2018; Lu et al., 2019; McGreevy et al., 2023). Based on the residuals of the regression of each DNAm ageing clock on chronological age, age‐adjusted estimates of DNAmAgeAccel, namely, HorvathAgeAccel (years), HannumAgeAccel (years), PhenoAgeAccel (years), GrimAgeAccel (years), and FitAgeAccel (years) were calculated.

### 5.9 Statistical analysis

As descriptive data, continuous variables are presented as mean ± standard deviation (SD) and categorical variables are presented as the number of persons and percentages (Table 1 and Table S2). Pearson's correlation analysis was performed for the relationships between chronological age and each DNAm ageing clock, and between each DNAmAgeAccel, and correlation coefficients (*r*) were presented. The statistical power of the correlation analysis was 0.80, assuming a sample size of 144 and an *r* value of 0.230. The relationship between lifestyle‐related variables and DNAmAgeAccel was examined via partial correlation analysis adjusted by chronological age, as well as smoking and drinking status, and *r* values were presented. In this study, 13 variables were extracted for further analysis, which had significant correlations and met the criteria of *r* ≤ −0.2 or *r* ≥ 0.2 as a result of the partial correlation analysis. In addition, the relationship between smoking pack years and each DNAmAgeAccel was performed through partial correlation analysis adjusted by chronological age, and *r* values were presented. The statistical powers of the partial correlation analysis were 0.610 and 0.805, respectively, assuming a sample size of 126 and an *r* value of 0.200 and 0.250. The extracted variables were assigned to two groups based on clinical cutoffs or reference values, and ANCOVA was performed, adjusted by chronological age, as well as smoking and drinking status. More specifically, grip strength <28 kg, visceral fat ≥100 cm^2^, calf circumference < 34 cm, TG ≥150 mg/dL, and HDL‐C < 40 mg/dL were used as clinical cutoff values according to previous research (Chen et al., 2020; Teramoto et al., 2012). VO_2_/kg at VT and VO_2_/kg at Peak adopted the reference values reported in the previous studies, that is, 13.4 mL/kg/min and 22.7 mL/kg/min, respectively (Kaminsky et al., 2022; Vainshelboim et al., 2020). CHO < 50% of total energy intake, Cu < 90 mg, and vitamin C < 100 mg were used as the criteria for classification according to the Japanese Dietary Reference Intakes (The Ministry of Health, Labour and Welfare of Japan, 2020). Note that only two participants fell below the recommended value of *β*‐carotene (850 μg), thus the median value was used to divide the participants into two groups. The data for fat‐free mass and head fat percentage were divided into two groups by the median values. ANCOVA, adjusted by chronological age, was also used to compare categorical variables between groups. The results of these ANCOVAs are presented as means ±95% confidence intervals. The statistical power of ANCOVA is shown in Tables S3–S5, respectively.

Three multiple regression models were developed to identify the determinants of each DNAm ageing clock, and the standard partial regression coefficients and 95% confidence intervals for the independent variables were calculated. The dependent variables in each model were PhenoAgeAccel, GrimAgeAccel, and FitAgeAccel. Note that HorvathAgeAccel and HannumAgeAccel, which had no variables corresponding to *r* ≤ −0.2 or *r* ≥ 0.2 in the partial correlation analysis, were excluded from the analysis. The candidate independent variables in each model were VO_2_/kg at VT (mL/kg/min), VO_2_/kg at Peak (mL/kg/min), Grip strength (kg), fat‐free mass (kg), visceral fat area (cm^2^), calf circumference (cm), Head fat (%), TG (mg/dL), HDL‐C (mg/dL), CHO (g/1000 kcal/day), Cu (mg/1000 kcal/day), vitamin C (mg/1000 kcal/day), *β*‐carotene (*μ*g/1000 kcal/day), smoking (non‐smoker, past smoker, or current smoker), drinking (0–1 times a week, 2–4 times a week, or 5–7 times a week), and chronotype (moderate and definite morning type or intermediate and moderate evening type). The enter method was used to develop the model, and before using this method, we calculated the variance inflation factor (VIF) for each variable to avoid multicollinearity and to ensure that the VIF was less than 10. A two‐sided *p* value of less than 5% was expressed as statistically significant. The statistical power of the multiple regression analysis was 0.959, 1.000 assuming a sample size of 144, a multiple correlation coefficient (R) of 0.430 and 0.567 and *p* value of 0.05. All statistical analyses were performed using SPSS Statistics version 26 (IBM Corporation, Chicago, IL, USA).

## AUTHOR CONTRIBUTIONS

T.K., Z.R., and K.T. conceptualised and designed the study. S.S., K.O., M.H., I. M., and K.T. conceived and supervised the study. T.K., H.T., N.N., R.K., T.I., C.U., H‐K.K., K.S., K.I., S.S., and K.T. conducted the investigation. KM.M. and S.H. calculated the DNA methylation age and age acceleration. T.K., H.A., and K.T. performed statistical analyses and created the figures. T.K., Z.R., M.J., F.T., M.M., and K.T. interpreted the data. T.K. wrote the manuscript, and all authors read and agreed to the published version of the manuscript.

## FUNDING INFORMATION

This research was supported by Grant‐in‐Aid for Early‐Career Scientists (20 K19520) from the Japan Society for the Promotion of Science, and Grant for Special Research Projects from Waseda University (2020–410). This project is a collaborative research project with the Institute of Stress Science, Public Health Research Foundation.

## CONFLICT OF INTEREST STATEMENT

The authors declare that they have no conflict of interest.

## Supporting information


Appendix S1

## Data Availability

The measurement data used to support the findings of this study is available from the corresponding author upon reasonable request.
