# Supplementary material for: Associations between cardiorespiratory fitness and lifestyle‐related factors with DNA methylation‐based ageing clocks in older men: WASEDA'S Health Study
Source: Aging Cell. 2023 Aug 16;23(1):e13960. doi: 10.1111/acel.13960 (PMC10776125; doi:10.1111/acel.13960)
Supplement: Supplementary file 1 — Appendix S1 [file ACEL-23-e13960-s001.docx]

Table S1. List of previous studies on the relationship between physical activity, physical fitness, and DNAm ageing clocks

| **Publication information** | **Subjects** | **Related factors** | **Results** |
| --- | --- | --- | --- |
| Quach et al., 2017 | WHI: *n* = 3583, women, 50–79 yrs  InCHIANTI: *n* = 402, men and women, 30–100 yrs | PA (self-administered questionnaires and baseline interview) | There is a slight significant negative correlation between PA and EEAA, but no significant correlation with IEAA. |
| Gale et al., 2018 | LBC 1936: *n* = 248, men and women, 79 yrs | PA (accelerometer) | There is no significant correlation between PA and IEAA and EEAA, respectively. |
| Sillanpää et al., 2019 | FTC: *n* = 1249, men and women, 20–72 yrs  7 MZ pair, 9 DZ pair, (5 men, 11 women), 60.4 yrs | LTPA (reported/interviewed) | Among the twin pairs with LTPA discordance, DNAmAgeHorvath did not differ between active and inactive co-twins. This result was also similar in the subgroup analysis of MZ and DZ twins. |
| Kankaanpää et al., 2021 | The FinnTwin12 study Cohort:  FTC:  *n* = 326, men and women, MZ Twins, 21–25 yrs  *n* = 244, men and women, DZ Twins, 21–25 yrs  *n* = 294, men and women, MZ Twins, 55–72 yrs  *n* = 176, men and women, DZ Twins, 55–72 yrs | LTPA and OPA (reported/interviewed) | LTPA was associated with slower and OPA with faster GrimAgeAccel. However, this association weakened after adjusting the model for smoking status. |
| Sillanpää et al., 2021 | FTC:  ERMA Cohort:  *n* = 93, men and women, Young, 23–42 yrs  *n* = 47, women, Middle, 48–55 yrs  *n* = 46, men and women, Older, 57–69 yrs | PA (reported/accelerometer), VO_2max_ (GXT using a cycle ergometer) | PA is positively associated with HorvathAgeAccel and GrimAgeAccel in the skeletal muscle, respectively.  VO_2max_ and HorvathAgeAccel and GrimAgeAccel in the skeletal muscle are not significantly related after adjustment for sex and smoking. |
| Kresovich et al., 2021 | US nationwide Sister Study Cohort:  *n* = 2758, women, 35–75 yrs | PA (interviewed) | PA was negatively correlated with GrimAgeAccel after adjustment for covariates and BMI. |
| Spartano et al., 2023 | FHS: *n* = 2435, 54.9±14.3 yrs, men and women | PA (accelerometer) | PA (step per day and MVPA) were associated with lower GrimAgeAccel, which was partially explained by BMI. |
| Fox et al., 2023 | The Rhineland Cohort:  *n* = 3567, men and women, 30–94 yrs | PA (accelerometer) | After adjustment for age, sex, season, education, smoking, cell proportions, and batch effects, PA (e.g., step counts, MET-Hours and % Time spent in MVPA) was negatively associated with PhenoAgeAccel and GrimAgeAccel, respectively. |
| Jokai et al., 2023 | Volunteers in the WRMR:  *n* = 303, men and women, 33–88 yrs | Indirectly calculated VO_2max_ (Chester step test) | The DNAmFitAge of high-fitness individuals was significantly lower than that of low/medium-fitness individuals. |

WHI, The Woman’s Health Initiative; InCHIANTI, The Invecchiare nel Chianti; LBC, The Lothian Birth Cohort 1936; FTC, Finnish Twin Cohort (includes three cohort studies: (1) The older twin cohort of twins born before 1958, (2) The Finntwin16, born in 1975–1979, and (3) The Finntwin12, born in 1983–1987); ERMA, Estrogen Regulation of Muscle Apoptosis study; FHS, The Framingham Heart Study; WRMR, World Rowing Masters Regatta in Velence, Hungary; MZ, Monozygotic twin; DZ, Dizygotic twin; PA, physical activity; LTPA, Leisure time physical activity; OPA, occupational physical activity; MVPA, Moderate to vigorous physical activity; Vo_2max_, maximal oxygen uptake; GXT, Graded maximal exercise test; EEAA, extrinsic epigenetic age acceleration; IEAA, intrinsic epigenetic age acceleration.

Table S2. Participant characteristics in categorical variables

|  | ***n* (%)** | |
| --- | --- | --- |
| ***Smoking status*** | **144** |  |
| Non-smoker | 44 | (30.6) |
| Past smoker | 90 | (62.5) |
| Current smoker | 10 | (6.9) |
| ***Drinking status*** | **144** |  |
| 0–1 times/week | 41 | (28.5) |
| 2–4 times/week | 27 | (18.7) |
| 5–7 times/week | 76 | (52.8) |
| ***Sleep disorders*** | **143** |  |
| Yes | 43 | (30.0) |
| No | 100 | (70.0) |
| ***Chronotype*** | **141** |  |
| Moderate and obvious morning type | 83 | (58.9) |
| Intermediate and moderate evening type | 58 | (41.1) |
| ***Dyslipidemia*** | **144** |  |
| Yes | 61 | (42.4) |
| No | 83 | (57.6) |
| ***Hypertension*** | **144** |  |
| Yes | 68 | (47.2) |
| No | 76 | (52.8) |
| ***Diabetes*** | **144** |  |
| Yes | 20 | (13.9) |
| No | 124 | (86.1) |
| ***Lifestyle-related diseases*** | **144** |  |
| Yes | 97 | (67.4) |
| No | 47 | (32.6) |

Data are numbers (*n*) and percentages (%).

Table S3. DNA methylation age acceleration for each continuous variable by analysis of covariance

| **Variables** | **Classification** | **HorvathAgeAccel** | ***p*** | **HannumAgeAceel** | ***p*** | **PhenoAgeAccel** | ***p*** | **GrimAgeAccel** | ***p*** | **FitAgeAccel** | ***p*** |
| --- | --- | --- | --- | --- | --- | --- | --- | --- | --- | --- | --- |
|  |  |  | **Power** |  | **Power** |  | **Power** |  | **Power** |  | **Power** |
| VO_2max_**/kg at VT (mL/kg/min)** | Low (n = 45) | -0.845 (-2.160, 0.471) | *0.162* | 0.464 (-0.605, 1.533) | *0.256* | -0.171 (-1.759, 1.416) | *0.971* | 0.840 (0.008, 1.672) | ***0.011*** | -0.356 (-1.293, 0.581) | *0.624* |
| <=13.4 or 13.4< | High (n = 97) | 0.288 (-0.607, 1.182) | *0.287* | -0.283 (-1.009, 0.444) | *0.205* | -0.136 (-1.216, 0.943) | *0.050* | -0.466 (-1.032. 0.099) | *0.721* | -0.074 (-0.711, 0.563) | *0.078* |
| VO_2max_**/kg at Peak (mL/kg/min)** | Low (n = 32) | -1.098 (-2.661, 0.465) | *0.118* | 0.579 (-0.714, 1.872) | *0.317* | 0.763 (-1.171, 2.696) | *0.378* | 0.942 (-0.058, 1.942) | ***0.037*** | 0.378 (-0.743, 1.499) | *0.321* |
| <=22.7 or 22.7< | High (n = 112) | 0.314 (-0.520, 1.148) | *0.346* | -0.165 (-0.856, 0.525) | *0.169* | -0.218 (-1.250, 0.814) | *0.142* | -0.269 (-0.803, 0.265) | *0.554* | -0.262 (-0.860, 0.336) | *0.167* |
| **Grip strength (kg)** | Low (n = 13) | -1.495 (-3.995, 1.005) | *0.214* | -0.831 (-2.896, 1.234) | *0.406* | -1.75 (-4.818, 1.319) | *0.251* | 0.745 (-0.840, 2.331) | *0.309* | 0.667 (-1.110, 2.444) | *0.345* |
| <28 or 28<= | High (n = 130) | 0.163 (-0.617, 0.943) | *0.236* | 0.083 (-0.562, 0.727) | *0.131* | 0.131 (-0.826, 1.088) | *0.209* | -0.115 (-0.610, 0.379) | *0.174* | -0.228 (-0.782, 0.326) | *0.156* |
| **Fat-free mass (kg)** | Low (n = 74) | 0.322 (-0.714, 1.358) | *0.382* | 0.148 (-0.707, 1.002) | *0.627* | 0.542 (-0.730, 1.813) | *0.231* | 0.380 (-0.283, 1.044) | *0.108* | 0.700 (-0.015, 1.415) | ***0.002*** |
| Median | High (n = 70) | -0.341 (-1.406, 0.725) | *0.141* | -0.156 (-1.035, 0.723) | *0.077* | -0.573 (-1.880, 0.735) | *0.223* | -0.402 (-1.084, 0.280) | *0.362* | -0.986 (-1.722, -0.251) | *0.895* |
| **Visceral fat area (cm^2^)** | Low (n = 82) | -0.184 (-1.181, 0.814) | *0.682* | -0.280 (-1.109, 0.549) | *0.238* | -1.161 (-2.355, 0.034) | ***0.003*** | -0.415 (-1.050, 0.219) | ***0.037*** | -0.429 (-1.143, 0.286) | *0.146* |
| <100 or 100<= | High (n = 57) | 0.147 (-1.057, 1.351) | *0.069* | 0.512 (-0.488, 1.513) | *0.218* | 1.736 (0.295, 3.178) | *0.848* | 0.665 (-0.101, 1.431) | *0.554* | 0.413 (-0.449, 1.275) | *0.306* |
| **Calf circumference (cm)** | Low (n = 10) | -0.079 (-2.937, 2.780) | *0.968* | 1.135 (-1.211, 3.481) | *0.319* | 1.513 (-2.015, 5.042) | *0.374* | 1.715 (-0.105, 3.534) | ***0.048*** | 2.115 (0.126, 4.104) | ***0.018*** |
| <34 or 34<= | High (n = 126) | -0.138 (-0.943, 0.666) | *0.050* | -0.097 (-0.757, 0.564) | *0.168* | -0.139 (-1.132, 0.855) | *0.143* | -0.193 (-0.705, 0.320) | *0.509* | -0.384 (-0.944, 0.176) | *0.661* |
| **Head fat percentage (%)** | Low (n = 72) | -0.026 (-1.092, 1.040) | *0.947* | 0.330 (-0.544, 1.204) | *0.301* | 0.670 (-0.632, 1.972) | *0.159* | 0.073 (-0.614, 0.760) | *0.770* | 0.123 (-0.636, 0.882) | *0.380* |
| Median | High (n = 72) | 0.026 (-1.040, 1.092) | *0.051* | -0.330 (-1.204, 0.544) | *0.178* | -0.670 (-1.972, 0.632) | *0.291* | -0.073 (-0.760, 0.614) | *0.06* | -0.362 (-1.121, 0.396) | *0.141* |
| **TG (mg/dL)** | Low (n =118) | 0.013 (-0.810, 0.836) | *0.943* | -0.169 (-0.843, 0.506) | *0.256* | -0.547 (-1.537, 0.443) | ***0.013*** | -0.277 (-0.797, 0.242) | ***0.016*** | -0.407 (-0.984, 0.170) | ***0.025*** |
| <150 or 150<= | High (n = 26) | -0.059 (-1.839, 1.721) | *0.051* | 0.765 (-0.693, 2.224) | *0.205* | 2.481 (0.340, 4.622) | *0.706* | 1.258 (0.134, 2.381) | *0.677* | 1.184 (-0.063, 2.432) | *0.617* |
| **HDL-C (mg/dL)** | Low (n = 5) | -0.214 (-4.260, 3.832) | *0.915* | 0.337 (-2.993, 3.667) | *0.839* | 4.326 (-0.595, 9.248) | *0.079* | 1.869 (-0.719, 4.457) | *0.149* | 1.298 (-1.579, 4.176) | *0.323* |
| <40 or 40<= | High (n =139) | 0.008 (-0.748, 0.763) | *0.051* | -0.012 (-0.634, 0.610) | *0.055* | -0.156 (-1.074, 0.763) | *0.419* | -0.067 (-0.550, 0.416) | *0.303* | -0.171 (-0.708, 0.367) | *0.166* |
| **CHO (%)** | Low (n =81) | -0.214 (-1.227, 0.800) | *0.542* | -0.363 (-1.193, 0.467) | *0.208* | -0.011 (-1.259, 1.237) | *0.980* | 0.314 (-0.336, 0.963) | *0.164* | 0.228 (-0.491, 0.948) | *0.163* |
| <50 or 50<= | High (n =63) | 0.275 (-0.882, 1.432) | *0.052* | 0.466 (-0.482, 1.415) | *0.149* | 0.014 (-1.411, 1.439) | *0.050* | -0.403 (-1.145, 0.338) | *0.113* | -0.567 (-1.388, 0.254) | *0.120* |
| **Cu (mg/day)** | Low (n =14) | -1.000 (-3.400, 1.401) | *0.388* | 0.847 (-1.128, 2.822) | *0.374* | 1.645 (-1.301, 4.591) | *0.248* | 1.412 (-0.119, 2.943) | *0.057* | 0.730 (-0.982, 2.441) | *0.304* |
| <0.9 or 0.9<= | High (n =130) | 0.108 (-0.672, 0.887) | *0.054* | -0.091 (-0.733, 0.550) | *0.081* | -0.177 (1.134, 0.780) | *0.385* | -0.152 (-0.649, 0.345) | *0.139* | -0.211 (-0.767, 0.345) | *0.146* |
| **Vitamin C (mg//day)** | Low (n =30) | -0.110 (-1.781, 1.561) | *0.885* | 0.670 (-0.700, 2.039) | *0.282* | 1.508 (-0.528, 3.543) | *0.104* | 1.030 (-0.029, 2.089) | ***0.034*** | 0.247 (-0.943, 1.437) | *0.498* |
| <100 or 100<= | High (n =114) | -0.029 (-0.811, 0.869) | *0.081* | -0.176 (-0.865, 0.512) | *0.054* | -0.397 (-1.420, 0.626) | *0.156* | -0.271 (-0.803, 0.261) | *0.498* | -0.216 (-0.815, 0.382) | *0.185* |
| ***β*-carotene (*µ*g/day)** | Low (n =72) | 0.497 (-0.596, 1.590) | *0.225* | 0.564 (-0.331, 1.459) | *0.093* | 1.074 (-0.255, 2.403) | ***0.032*** | 0.455 (-0.245, 1.156) | *0.083* | 0.277 (-0.501, 1.056) | *0.174* |
| Median | High (n =72) | -0.497 (-1.590, 0.596) | *0.228* | -0.564 (-1.459, 0.331) | *0.389* | -1.074 (-2.403, 0.255) | *0.576* | -0.455 (-1.156, 0.245) | *0.410* | -0.517 (-1.295, 0.262) | *0.274* |

Data are means ± 95% confidence intervals (CI) adjusted by chronological age, smoking and drinking status. Bolded letters indicate that the results are statistically significant. Vo_2max_, oxygen uptake; VT, ventilatory threshold; TG, triglyceride; HDL-C, high-density lipoprotein cholesterol; CHO, carbohydrate; Cu, copper.

Table S4. DNA methylation age acceleration for each categorical variable by analysis of covariance

| **Variables** | **Classification** | **HorvathAgeAccel** | ***p*** | **HannumAgeAceel** | ***p*** | **PhenoAgeAccel** | ***p*** | **GrimAgeAccel** | ***p*** | **FitAgeAccel** | ***p*** |
| --- | --- | --- | --- | --- | --- | --- | --- | --- | --- | --- | --- |
|  |  |  | **Power** |  | **Power** |  | **Power** |  | **Power** |  | **Power** |
| **Smoking** | Non-smoker (n = 44) | 0.014 (-1.322, 1.349) | *0.845* | 0.224 (-0.876, 1.324) | *0.876* | 0.177 (-1.467, 1.822) | *0.763* | -1.241 (-2.101, -0.381) | ***<0.001*** | -0.955 (-1.912, 0.003) | ***0.010*** |
|  | Past smoker (n = 90) | 0.081 (-0.853, 1.016) | *0.076* | -0.076 (-0.846, 0.694) | *0.070* | -0.206 (-1.356, 0.945) | *0.092* | 0.117 (-0.484, 0.719) | *0.999* | -0.001 (-0.671, 0.668) | *0.790* |
|  | Current smoker (n = 10) | -0.792 (-3.611, 2.027) |  | -0.301 (-2.623, 2.022) |  | 1.071 (-2.399, 4.541) |  | 4.405 (2.591, 6.220) |  | 2.490 (0.469, 4.510) |  |
| **Drinking** | 0-1 times a week (n = 41) | -0.051 (-1.452, 1.351) | *0.953* | -0.137 (1.289, 1.014) | *0.700* | -0.389 (-2.113, 1.334) | *0.745* | -0.999 (-1.965, -0.032) | ***0.013*** | -1.002 (-2.024, 0.021) | *0.131* |
|  | 2-4 times a week (n = 27) | 0.242 (-1.474, 1.957) | *0.057* | 0.544 (-0.865, 1.953) | *0.106* | -0.352 (-2.461, 1.757) | *0.096* | -0.503 (-1.686, 0.679) | *0.759* | 0.080 (-1.172, 1.331) | *0.418* |
|  | 5-7 times a week (n - 76) | -0.059 (-1.077, 0.960) |  | -0.119 (-0.956, 0.718) |  | 0.335 (-0.917, 1.588) |  | 0.718 (0.015, 1.420) |  | 0.285 (-0.458, 1.029) |  |
| **Sleep disorders** | No (n = 100) | -0.260 (-1.140, 0.620) | *0.363* | -0.170 (-0.899. 0.559) | *0.398* | -0.099 (-1.192, 0.993) | *0.736* | 0.022 (-0.609, 0.653) | *0.922* | -0.076 (-0.733, 0.580) | *0.852* |
|  | Yes (n = 43) | 0.480 (-0.862, 1.822) | *0.148* | 0.400 (-0.711, 1.512) | *0.134* | 0.241 (-1.425, 1.908) | *0.063* | -0.035 (-0.997, 0.927) | *0.051* | -0.190 (-1.191, 0.811) | *0.054* |
| **Chronotype** | Intermediate and moderate evening type (n = 58) | -0.449 (-1.601, 0.702) | *0.449* | -0.263 (-1.197, 0.672) | *0.523* | 0.457 (-0.932, 1.845) | *0.291* | 0.652 (-0.164, 1.467) | ***0.033*** | 0.427 (-0.421, 1.267) | *0.071* |
|  | Moderate and definite morning type (n = 83) | 0.127 (-0.835, 1.089) | *0.117* | 0.132 (-0.649, 0.913) | *0.097* | -0.513 (-1.674, 0.647) | *0.183* | -0.505 (-1.187, 0.177) | *0.570* | -0.590 (-1.295, 0.116) | *0.439* |

Data are means ± 95% confidence intervals (CI) adjusted by chronological age. Bolded letters indicate that the results are statistically significant.

Table S5. DNA methylation age acceleration for each disease by analysis of covariance

| **Variables** | **Classification** | **HorvathAgeAccel** | ***p*** | **HannumAgeAceel** | ***p*** | **PhenoAgeAccel** | ***p*** | **GrimAgeAccel** | ***p*** | **FitAgeAccel** | ***p*** |
| --- | --- | --- | --- | --- | --- | --- | --- | --- | --- | --- | --- |
|  |  |  | **Power** |  | **Power** |  | **Power** |  | **Power** |  | **Power** |
| **Dyslipidaemia** | No (n = 83) | -0.306 (-1.273, 0.661) | *0.338* | -0.461 (-1.251, 0.329) | ***0.079*** | -0.584 (-1.770, 0.601) | *0.137* | -0.792 (-1.451, -0.132) | ***<0.001*** | -0.903 (-1.593, -0.214) | ***< 0.001*** |
|  | Yes (n = 61) | 0.417 (-0711, 1.545) | *0.159* | 0.627 (-0.295, 1.549) | *0.070* | 0.795 (-0.588, 2.178) | *0.318* | 1.077 (0.308. 1.847) | *0.952* | 0.947 (0.142, 1.751) | *0.929* |
| **Hypertension** | No (n = 76) | 0.527 (-0.480, 1.533) | *0.135* | 0.186 (-0.649, 1.020) | *0.523* | 0.180 (-1.069, 1.429) | *0.680* | -0.361 (-1.077, 0.355) | *0.149* | -0.341 (-1.090, 0.408) | *0.397* |
|  | Yes (n = 68) | -0.589 (-1.653, 0.476) | *0.321* | -0.208 (-1.090, 0.675) | *0.106* | -0.201 (-1.522, 1.120) | *0.070* | 0.404 (-0.354, 1.161) | *0.302* | 0.128 (-0.664, 0.920) | *0.135* |
| **Diabetes** | No (n = 124) | -0.061 (-0.854, 0.733) | *0.688* | -0.039 (-0.693, 0.615) | *0.753* | -0.299 (-1.268, 0.670) | *0.106* | -0.120 (-0.682, 0.443) | *0.263* | -0.315 (-0.897, 0.266) | *0.077* |
|  | Yes (n = 20) | 0.376 (-1.609, 2.360) | *0.069* | 0.242 (-1.393, 1.877) | *0.134* | 1.853 (-0.570, 4.277) | *0.366* | 0.741 (-0.664, 2.147) | *0.200* | 1.094 (-0.359, 2.548) | *0.423* |
| **Lifestyle-related diseases** | No (n = 47) | 0.059 (-1.230, 1.348) | *0.913* | -0.182 (-1.243, 0.879) | *0.680* | -0.507 (-2.092, 1.078) | *0.443* | -0.537 (-1.447, 0.374) | *0.158* | -0.745 (-1.690, 0.201) | *0.114* |
|  | Yes (n = 97) | -0.028 (-0.926, 0.869) | *0.051* | 0.088 (-0.651, 0.827) | *0.097* | 0.245 (-0.858, 1.349) | *0.119* | 0.260 (-0.374, 0.894) | *0.292* | 0.183 (-0.475, 0.841) | *0.353* |

Data are means ± 95% confidence intervals (CI) adjusted by chronological age. Bolded letters indicate that the results are statistically significant.

**Figure S1. Partial correlation coefficients between expiratory gas parameters and DNA methylation age acceleration.** Data represent partial correlation coefficients (*r*) adjusted by chronological age, smoking, and drinking status (chronological age was adjusted by smoking and drinking status). Red cells indicate positive and blue cells indicate negative correlations. PETO_2_, end-tidal oxygen concentration; PETCO_2_ end-tidal carbon dioxide concentration, VE/ VO_2max_; ventilatory equivalent for oxygen; VE/VCO_2_; ventilatory equivalent for carbon dioxide; VD/VT, deadspace volume to tidal volume ratio; VT, ventilatory threshold; RR, respiratory rate; VO_2max_, oxygen uptake; respiratory quotient; HR, heart rate; VE vs VCO_2_ slope, ventilation to carbon dioxide production. Significant correlations at *p* < 0.05, *p* < 0.01, and *p* < 0.001 are indicated by *, **, and ***, respectively.

**Figure S2. Partial correlation coefficients between anthropometric variables and DNA methylation age acceleration.** Data represent partial correlation coefficients (*r*) adjusted by chronological age, smoking and drinking status (chronological age was adjusted by smoking and drinking status). Red cells indicate positive and blue cells indicate negative correlations. BMI, body mass index. Significant correlations at *p* < 0.05, *p* < 0.01, and *p* < 0.001 are indicated by *, and **, respectively.

**Figure S3. Partial correlation coefficients between body composition by dual-energy X-ray absorptiometry and DNA methylation age acceleration.** Data represent partial correlation coefficients (*r*) adjusted by chronological age, smoking and drinking status (chronological age was adjusted by smoking and drinking status). Red cells indicate positive and blue cells indicate negative correlations. Significant correlations at *p* < 0.05 are indicated by *.

**Figure S4. Partial correlation coefficients between blood biochemical parameters and DNA methylation age acceleration.** Data represent partial correlation coefficients (*r*) adjusted by chronological age, smoking and drinking status (chronological age was adjusted by smoking and drinking status). Red cells indicate positive and blue cells indicate negative correlations. HbA1c, hemoglobin A1c, TG, triglyceride; Total-C, total-cholesterol; HDL-C, high-density lipoprotein cholesterol; LDL-C, low-density lipoprotein cholesterol. Significant correlations at *p* < 0.05 and *p* < 0.01 are indicated by * and **, respectively.

**Figure S5. Partial correlation coefficients between nutrient intake variables and DNA methylation age acceleration.** Data represent partial correlation coefficients (*r*) adjusted by chronological age, smoking and drinking status (chronological age was adjusted by smoking and drinking status). Red cells indicate positive and blue cells indicate negative correlations. CHO, carbohydrate; Fe, iron; Zn, zinc; Cu, copper; Mn, manganese. Significant correlations at *p* < 0.05, *p* < 0.01, and *p* < 0.001 are indicated by *, **, and ***, respectively.
